# Pilomotor Seizures in a Patient With LGI1 Encephalitis

**DOI:** 10.3389/fneur.2020.00061

**Published:** 2020-02-20

**Authors:** Jinxia Yang, Qiying Sun, Guang Yang

**Affiliations:** ^1^Department of Geriatrics, Xiangya Hospital, Central South University, Changsha, China; ^2^Department of Emergency, Xiangya Hospital, Central South University, Changsha, China

**Keywords:** limbic encephalitis, anti-LGI1 antibody, faciobrachial dystonic seizures, pilomotor seizures, case report

## Abstract

Limbic encephalitis (LE) with antibodies against leucine-rich glioma inactivated protein 1 (LGI1) is an autoimmune disease with variable clinical features, including seizures, cognitive disorders, psychiatric disturbances, and hyponatremia. The majority of these patients present faciobrachial dystonic seizures (FBDS), which are regarded as a characteristic symptom. A few cases have reported pilomotor seizures as the main manifestation of anti-LGI1 encephalitis. Here, we described a Chinese woman with frequent pilomotor seizures who was finally diagnosed as having anti-LGI1 encephalitis. Our report emphasizes the possible significance of pilomotor seizures in anti-LGI1 encephalitis.

## Introduction

Since firstly reported in 2010, limbic encephalitis (LE) with antibodies against leucine-rich glioma inactivated protein 1 (LGI1) has been an important part of autoimmune encephalitis, which often manifests as variable clinical features, including seizures, cognitive impairment, psychiatric disturbances, and hyponatremia. The seizures include typical seizure events and more distinctive semiologies such as faciobrachial dystonic seizures (FBDS), piloerection, and bradycardia. The majority of patients present FBDS, which is regarded as the characteristic symptom in anti-LGI1 encephalitis. Ictal piloerection (IP) was commonly considered to be an autonomic epilepsy, and has been reported in many neurological diseases, especially in LE (1). Many researches have suggested pilomotor seizures as a specific section of multiple seizures associated with LE (2, 3), but reports that patient with LGI1 encephalitis only present pilomotor seizures are rare (4). In this report, we described a Chinese woman with frequent pilomotor seizures who was finally diagnosed as anti-LGI1 encephalitis.

## Case Report

**Figure 1:**
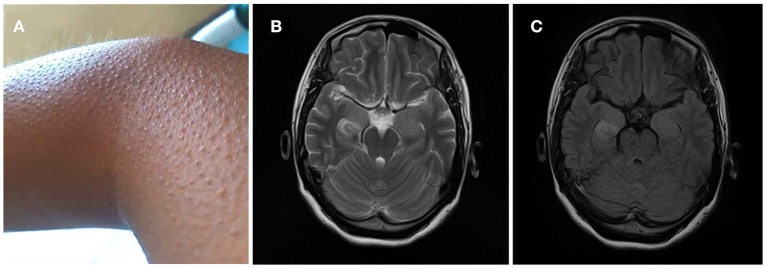
**(A)** Frequent piloerection involved the patient's limbs, lasted from a few seconds to >2 min, and occurred several times throughout the day. **(B,C)** MRI T2-weighted and flair images showed signal change on right hippocampus.

A 32-year-old, previously healthy, right-handed woman without personal or family history of epilepsy or neurological diseases was admitted in 2018 because of frequent piloerection (Figure 1A). The first episode developed in May after overwork. The pilomotor involved four limbs, lasted from a few seconds to >2 min, and occurred several times throughout the day. These episodes were either isolated or were associated with tachypnea and tachycardia, but there were no fever, headache, tremor, clonus, edema, loss of awareness, remarkable memory impairment, or psychiatric disorders. Three weeks after the first episode, she presented to the outpatient clinics of local hospital with repeated piloerection. Brain computed tomography (CT) and EEG were normal; therefore, the patient refused hospitalization, and received no medication. Given the increasing frequency of pilomotor seizures, she was admitted to another local hospital in mid-June. Cerebrospinal fluid (CSF) analysis was normal; brain MRI revealed T2 hyperintensity on bilateral hippocampus. Three days after admission, a diagnosis of possible viral encephalitis was established and the empirical treatment with antiviral drugs started. However, the patient was discharged home without any improvement after being treated for 5 days, and progressively the frequency of piloerection increased to dozens of times per day. After discharge, she also visited our outpatient clinic specialized in epilepsy and was given a trial of antiepileptic drugs (carbamazepine 0.1 g bid) for almost 1 month for the recurrent piloerection, but the frequency of pilomotor seizures still increased.

**Figure 2:**
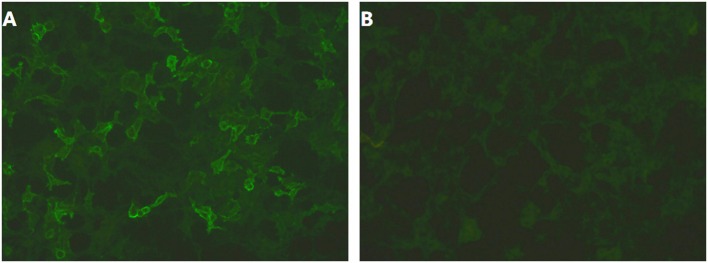
**(A,B)** Autoimmune encephalitis antibody testing showed positive antibodies against LGI1 in the patient's serum (++, 1:32), but negative in CSF.

Two months after onset, she was admitted to our hospital. On admission, examination showed that her cognition was normal with Mini-Mental State Examination: 26/30 (with the diploma of primary school). Blood testing include routine blood tests, blood glucose, blood lipids, hepatic and renal function testing, serum electrolytes, coagulation studies, HIV antibodies, hepatitis B and C, syphilis serology, thyroid serologic testing, ANCA, systemic autoimmune antibodies, anti-GAD antibodies, antineuronal antibodies testing (anti-Hu, anti-Ri, anti-Yo, anti-MA2, anti-amphiphysin), which were all negative except mild leukocytosis and hypercholesterolemia (TC 5.74 mmol/L, LDL 3.9 mmol/L). Carbamazepine was switched to levetiracetam (0.5 g bid); despite these, the episodes were still frequent and occurred dozens of times per day. Two days after admission, 24-h continuous electroencephalography (EEG) demonstrated abnormal slow wave activity in bilateral brain during pilomotor, especially in the right frontal lobe and temporal lobe. MRI shows signal change on right hippocampus (Figures 1B,C). CSF analysis revealed normal cytology and chemistry. In addition, autoimmune encephalitis antibodies were detected by indirect immunofluorescence test through a kit (Euroimmun AG, Germany) containing HEK293 cells transfected with plasmids containing the NMDAR, GluR1/GluR2 subunits of the AMPAR, GABABR, LGI1, and CASPR2 in both serum and CSF. Three days after lumbar puncture, autoimmune encephalitis antibody testing showed positive antibodies against LGI1 in the patient's serum (++, 1:32), but negative in CSF (Figure 2).

Given the positive result of LGI1 antibodies, the patient was started on immunotherapy in the form of intravenous methylprednisolone pulse (500 mg/day), followed by halved doses for every 3 days. She made a significant recovery with decrease of frequency and duration of pilomotor seizures. The patient was discharged home on a reducing course of oral prednisone at an initial dose of 60 mg daily.

No pilomotor seizures or other neurologic manifestation were reported at the 3-months and 1-year follow-up.

## Discussion

A triad of cognitive impairment, hyponatremia, psychiatric disturbances, and various forms of epilepsy characterizes LGI-1 antibody encephalitis. FBDS are highly distinctive semiology of seizures in LGI1 encephalitis. Sidra Aurangzeb reported 16 patients with LGI1-antibody encephalitis, among which FBDS were recorded in 14 patients, suggesting the close association between FBDS and LGI1 antibodies (5). However, few case reports have suggested pilomotor seizures as a specific manifestation associated with LGI1 antibodies. Stephan Wieser reported a male patient who presented frequent piloerection with antibodies to voltage-gated potassium channels but was not confirmed to be positive for LGI1 antibodies (3). Rodrigo Rocamora reported three patients with piloerection attending for video telemetry in specialist epilepsy centers, which was associated with LGI1 antibodies (4). Compared to those cases, our patient did not show obvious cognitive impairment, or relatively characteristic hyponatremia.

Ictal piloerection is typically associated with seizures generated in the temporal lobe. Pilomotor activity associated with frontal origin has also been described (6). An early research showed that stimulation of the insula, hippocampus, amygdala, hypothalamus, midbrain, or medial prefrontal cortex leads to piloerection, supporting the view that the origin of ictal piloerection is within or close to the central autonomic network (7). A recent review shows IP was particularly associated with autoimmune encephalitis and high-grade glioma, suggesting IP's particular importance in diagnosis of LE (8). In our report, abnormal activity in bilateral brain during pilomotor, especially in the right frontal lobe and temporal lobe, may explain why this woman presents pilomotor seizures.

Seizures of LE with antibodies against LGI1 have poor response to antiepileptic drugs (AEDs) but respond well to immunotherapy (9). Those previous cases who presented pilomotor seizures as specific manifestation also showed remarkable improvement after immunoloregulation (3, 4). Similarly, the pilomotor seizures of our patient were refractory to AEDs and respond much better to glucocorticoids, which is consistent with previous researches.

## Conclusion

This case described a Chinese woman with frequent pilomotor seizures who was finally diagnosed as anti-LGI1 encephalitis. We emphasize the possible significance of pilomotor seizures in anti-LGI1 encephalitis, and recognition of this kind of seizures, and their association with LGI1 antibodies, should prompt timely initiation of immunotherapies.

## Data Availability Statement

All datasets generated for this study are included in the article/supplementary material.

## Ethics Statement

All clinical data in this case report were provided by the patient or collected by our team members with the patient's consent. There was no additional invasive test or experimental drugs used out of order for the patient. A written informed consent was obtained from the patient for the participation in the study and the publication of this report. The case report is exempt from institutional review board approval.

## Author Contributions

JY and GY acquired the clinical data, reviewed the literature, and drafted the manuscript. QS and GY designed the study, oversaw data acquisition, supervised the initial drafting, and critically revised the manuscript.

### Conflict of Interest

The authors declare that the research was conducted in the absence of any commercial or financial relationships that could be construed as a potential conflict of interest.
